# Curcumin Inhibits Hyperandrogen-Induced IRE1*α*-XBP1 Pathway Activation by Activating the PI3K/AKT Signaling in Ovarian Granulosa Cells of PCOS Model Rats

**DOI:** 10.1155/2022/2113293

**Published:** 2022-08-24

**Authors:** Yaling Zhang, Lei Wang, Yajing Weng, Daojuan Wang, Rong Wang, Hongwei Wang, Lihui Wang, Shanmei Shen, Hongwei Wang, Yan Li, Yong Wang

**Affiliations:** ^1^State Key Laboratory of Analytical Chemistry for Life Science & Jiangsu Key Laboratory of Molecular Medicine, Medical School, Nanjing University, Nanjing 210093, China; ^2^Department of Clinical Laboratory, Jiangsu Province Hospital on Integration of Chinese and Western Medicine, China; ^3^Danyang Hospital Affiliated to Nantong University, Danyang, Jiangsu 212300, China; ^4^Department of Endocrinology, The Affiliated Drum Tower Hospital, Medical School, Nanjing University, Nanjing 210093, China; ^5^Department of Obstetrics and Gynecology, Yancheng First Hospital of Nanjing University Medical School, Yancheng, China

## Abstract

**Background:**

Hyperandrogenism is a common characteristic of polycystic ovary syndrome (PCOS). Long-term, continuous exposure to hyperandrogenic environments may cause excessive endoplasmic reticulum (ER) stress in ovarian granulosa cells (GCs). Curcumin is a polyphenol extracted from turmeric rhizomes which has several pharmacological effects that may benefit patients with PCOS. To explore whether curcumin can inhibit hyperandrogen-induced ER stress in ovarian GCs of PCOS rats and to elucidate the possible underlying mechanisms.

**Methods:**

We developed PCOS model rats by exposure to hyperandrogenic conditions and divided the rats into control, PCOS, and PCOS+curcumin (200 mg/kg, for 8 weeks) groups. The levels of ER stress-related proteins and PI3K/AKT phosphorylation were measured in the ovarian tissue of all experimental groups by real-time quantitative PCR, western blotting, immunohistochemistry, and immunofluorescence. Subsequent in vitro analysis on primary cultured GCs was performed to confirm the influence of curcumin on ER stress inhibition by immunofluorescence and western blotting.

**Results:**

Curcumin protects GCs from hyperandrogen-induced apoptosis in PCOS model rats by inhibiting the ER stress-related IRE1*α*-XBP1 pathway and activating the PI3K/AKT signaling pathway.

**Conclusions:**

These observations indicate that curcumin might be a safe and useful supplement for PCOS patients.

## 1. Introduction

Polycystic ovary syndrome (PCOS) is a multifactorial endocrine and metabolic disorder that affects 10 to 18% of women of reproductive age [1]. It is characterized by chronic anovulation, hyperandrogenism, and polycystic ovaries [2, 3]. In addition, many patients with PCOS experience insulin resistance (IR), obesity, and low-grade chronic inflammation. Obesity and low-grade inflammation both contribute to the onset of IR, which might empower androgenic activity, while high levels of androgens may further blunt IR and worsen the clinical presentation of PCOS, which suggests that this vicious circle may play a critical role in the pathophysiology of PCOS [4, 5]. However, the cause of PCOS is not yet clear, and the underlying pathological mechanisms are complex. In recent years, lifestyle intervention has been listed as a first-line treatment for PCOS.

Curcumin is a fat-soluble polyphenol extracted from turmeric rhizomes. It is the main active component in turmeric and exerts pharmacological effects [6]. Significant research has shown that curcumin possesses potent biochemical and biological activities. Furthermore, curcumin has low toxicity and almost no adverse reactions [7–11]. Several in vivo and in vitro studies have confirmed that curcumin or turmeric extract can improve glucose metabolism in diabetes by alleviating IR [12, 13]. Studies have shown that 50%–70% of PCOS patients experience IR and hyperinsulinemia, and IR is closely related to PCOS hyperandrogenemia and ovulation dysfunction [14]. Nevertheless, whether curcumin can correct ovarian follicle development disorders in PCOS, and if so, by what molecular mechanism, remains unclear.

A growing number of studies have shown that excessive androgens activate endoplasmic reticulum (ER) stress in ovarian granulosa cells (GCs), eventually affecting oocyte maturation, follicle formation, and ovulation [15–17]. X-box binding protein 1 (XBP1), a nuclear transcription factor, is located downstream of IRE1*α*. The IRE1*α*-XBP1 signaling pathway is an evolutionarily conserved branch of the unfolded protein response (UPR) that maintains ER homeostasis while simultaneously governing various immune metabolic processes [18, 19]. However, the physiological consequences of the IRE1*α*-XBP1 signaling in GCs during PCOS ovarian dysfunction remain largely unexplored.

The phosphatidylinositol 3-kinase (PI3K) signaling pathway is a key pathway regulating cell proliferation, apoptosis, migration, metabolism, and other physiological and pathological processes [20, 21]. Recent studies have confirmed that the PI3K/AKT signaling pathway is involved in the recovery of ovarian function and regulation of the growth and apoptosis of GCs during follicular development [22–24]. However, the relationship between ER stress and the PI3K/AKT pathway in GCs of the PCOS ovary has not been elucidated. Therefore, we sought to determine whether curcumin could alleviate hyperandrogenism-induced ER stress in PCOS by activating the PI3K/AKT signaling pathway, and to elucidate the mechanism by which curcumin regulates GC apoptosis.

## 2. Materials and Methods

### 2.1. Rat PCOS Model and Experimental Protocol

Thirty female Sprague-Dawley (SD) rats (21 days old, weighing 50 ± 10 g) were purchased from Shanghai Xipuer-Bikai Laboratory Animal (Shanghai, China). Rats were randomly divided into three groups (*n* = 10 per group): normal control group (control), PCOS group (PCOS), and curcumin treatment group (PCOS+CUR). To induce the PCOS model, dehydroepiandrosterone (DHEA) (6 mg/100 g·d) dissolved in 0.2 mL sesame oil was administered by hypodermic injection for 35 consecutive days. Rats of the PCOS+CUR group were further administered curcumin (200 mg/kg) dissolved in 0.2 mL sesame oil by intragastric administration for 8 weeks. The rats in the normal control group received only sesame oil. This study was approved by the Institutional Research Animal Committee of Nanjing University, and all animal experiments were performed in accordance with the principles and guidelines of the Institutional Animal Care committee.

### 2.2. Culture of Primary GCs

Twenty-one-day-old female rats were injected with pregnant maternal serum gonadotropin (20 IU) 48 h in advance [25]. Rats were sacrificed by cervical dislocation and then soaked and disinfected in 75% alcohol for 20 min. Harvesting and isolation of the GCs were subsequently performed in a vertical flow clean bench. Cell debris in the GCs was removed using 200 *μ*m cell strainers. Primary GCs were cultured in Dulbecco's modified Eagle's medium/nutrient mixture F-12 (DMEM-F12) containing 10% fetal bovine serum (FBS; Wisent, Quebec, Canada) and 1% penicillin-streptomycin (Gibco, Waltham, MA, USA) in a 5% CO_2_ atmosphere and maintained at 37°C.

### 2.3. Estrous Cycle Detection

On the 21^st^ day of modeling, the phase of the estrous cycle was investigated by daily observations of vaginal smears for rats at 9.00 h for 10 days. A sterile cotton swab was gently inserted into the rat's vagina in and out three times, swabbing the vaginal wall, and then rotated onto clean glass slides, air dried, fixed with 70% ethanol, stained with methylene blue (Thomas Baker, Mumbai, India), and evaluated under a light microscope. Three types of cells were observed: small round cells (leukocytes), round and nucleated cells (epithelial cells), and irregular anucleated cells (cornified cells). The proportions of these three types of cells were used to determine the phase of the estrous cycle.

### 2.4. Physical Parameters and Hematoxylin and Eosin (H&E) Staining

The body weights of all the experimental groups were recorded every other day until the end of the modeling or curcumin treatment. After dissection, standard measurement methods were used to record the physical parameters, including abdominal fat and ovary weight. Ovarian and abdominal fat tissues were fixed in 10% formalin for histological analysis. Specimens were embedded in paraffin blocks, sectioned to approximately 5 *μ*m thickness, and stained with hematoxylin and eosin. The stained sections were observed under an optical microscope (Leica Microsystems, Germany).

### 2.5. Blood Sampling and Serum Hormone Measurement

After 8 weeks of treatment with curcumin, rats were anesthetized with pentobarbital sodium, and blood was collected by cardiac puncture. The blood samples were centrifuged (1000 rpm for 5 min), and the serum was separated and stored at -80°C for until the determination of testosterone (T), luteinizing hormone (LH), and follicle-stimulating hormone (FSH) levels using enzyme-linked immunosorbent assay (ELISA) (Elabscience Biotechnology, China).

### 2.6. Immunohistochemistry and Immunofluorescence

To evaluate protein localization and intensity, immunohistochemistry and immunofluorescence were performed according to previously described methods [26, 27]. Ovarian tissue slides were dewaxed in xylene and graded series of ethanol solutions. Slides were then fixed in 4% paraformaldehyde and blocked with 3% bovine serum albumin (BSA) at 37°C. The sections were incubated overnight at 4°C with antibodies against p-IRE1*α* (1: 200, ab124945, Abcam, Cambridge, UK), XBP1 (1: 100, WL00708, Wanleibio, Shenyang, China), p-PI3K (1 : 200, AF3242, Affinity, USA), p-AKT (1: 200, 4060T, CST, USA), p-p53 (1: 200, ab33889, Abcam, UK), p-NF-*κ*B (1: 200, 3033S, CST, USA), and cleaved-caspase-3 (1: 200, 66470-2-Ig, Proteintech, USA). All secondary antibodies were diluted (1 : 2000) and incubated at 25°C for 2 h. The sections were processed according to the avidin-biotinylated-peroxidase complex and DAB staining techniques, followed by observation under an optical microscope. Nuclei were counterstained with 4,6-diamidino-2-phenylindole (DAPI) (1 : 2000, C1002, Beyotime, China) for 30 min and photographed using an Olympus laser scanning confocal microscope (FV3000).

### 2.7. TUNEL Analysis

TUNEL assay was used to detect DNA damage. A fluorescein isothiocyanate (FITC) TUNEL Cell Apoptosis Detection Kit (G1501-100T, Servicebio, Wuhan, China) was used in accordance with the manufacturer's instructions. Slides treated with DNase I for 30 min served as the positive controls. DAPI was used to stain the nuclei.

### 2.8. CCK-8 Analysis of Cell Viability

Primary GCs were seeded in 96-well plates at a density of 1 × 10^5^/mL. Our previous research showed that different concentrations of dihydrotestosterone (DHT) (0, 0.1, 0.5, 1, 2, and 5 *μ*M) can induce GC apoptosis [27]. Therefore, we treated GCs with the optimal induction concentration (5 *μ*M) of DHT for 24 h and subsequently added different concentrations (5, 10, 15, and 20 *μ*M) of curcumin for 24 h. Cell counting kit-8 (CCK-8) solution (10 *μ*L; A311-02-AA, Vazyme Biotech, Nanjing, China) was added to each well, followed by incubation for 4 h at 37°C. The absorbance of each sample was measured using a microplate reader at a wavelength of 450 nm.

### 2.9. Intracellular Reactive Oxygen Species (ROS) Levels

Primary GCs were treated with DHT, hydrogen peroxide (H_2_O_2_), and different concentrations of curcumin (5, 10, and 20 *μ*M), and GCs were then loaded with 2,7-dichlorodihydrofluorescein diacetate (H2-DCFDA, 10 *μ*M) and incubated for 30 min at 37°C. The medium was discarded, and cells were washed with PBS three times on ice. Images of the cells were captured using an Olympus laser scanning confocal microscope (FV3000). Images were analyzed using the Image-Pro Plus 6.0.

### 2.10. Apoptosis Assay

GCs were seeded in 24-well plates at a concentration of 1 × 10^5^ cells/well and cultured in full culture medium for 48 h. Apoptosis of GCs was detected using the Annexin V-FITC apoptosis detection kit (C1062L, Beyotime, Shanghai, China). Briefly, after GCs were treated with specified concentrations of corresponding drug for specified time, they were labeled with Annexin V-FITC (5 *μ*L) and propidium iodide (PI) (10 *μ*L) for 15 min in the dark at room temperature. Green (Annexin V-FITC) and red (PI) fluorescence were examined using flow cytometry and an Olympus laser scanning confocal microscope (FV3000).

### 2.11. Quantitative Real-Time PCR (qRT-PCR)

Total RNA from ovarian tissue and GCs was extracted using TRIzol reagent (R401-01, 301, Vazyme Biotech, China), and a reverse transcription kit (R223-01, Vazyme Biotech, China) was used for cDNA synthesis. The primer sequences are listed in Table 1. Quantitative RT-PCR was performed with the ABI ViiA 7 Real-Time PCR system (ABI, USA) using the SYBR Green PCR Master Mix (Q441-02, Vazyme, China). Quantitative RT-PCR was performed according to Table 2. The expression levels were calculated using the 2^-*ΔΔ*^CT method.

### 2.12. Western Blot

Proteins from ovarian tissue and GCs were extracted using RIPA lysis buffer (P0013B, Beyotime, China) containing Halt™ Protease Inhibitor Cocktail (B14001, Selleck Chemicals, Houston, TX, USA). Total proteins (30 *μ*g/lane) were separated by 10% sodium dodecyl sulfate-polyacrylamide gel electrophoresis, and the protein bands were transferred onto polyvinylidene difluoride membranes (IPVH00010, Merck Millipore, St. Louis, MO, USA). The membranes were then blocked with Tris-buffered saline with Tween-20® containing 5% bovine serum albumin (Bio-Rad, Hercules, CA, USA) for 90 min at 25°C and subsequently incubated with the corresponding primary antibodies GRP-78 (1 : 1000, WL03157, Wanleibio, Shenyang, China), CHOP (1 : 1000, abs135545, Absin, Shanghai, China), IRE1*α* (1 : 1000, 27528-1-AP), p-IRE1*α* (1: 1000), XBP1 (1 : 500), p-PI3K (1: 1000), PI3K (1 : 1000, 4292S, CST, USA), p-AKT (1: 1000), AKT (1 : 1000, 4691T, CST, USA), p-p53 (1 : 1000), p-NF-*κ*B (1 : 1000), NF-*κ*B (1 : 1000, 8242S, CST, USA), p-JNK (1 : 1000, 4668S, CST, USA), JNK (1 : 1000, 9252T, CST, USA), Bad (1 : 1000, 67830-1-Ig, Proteintech), Bax (1 : 2000, 50599-2-Ig, Proteintech), Bcl-2 (1 : 1000, Wanleibio), caspase-12 (1 : 1000, 3282-30T, BioVision Inc., Milpitas, CA, USA), caspase-9 (1 : 1000, 10380-1-AP, Proteintech), and cleaved-caspase-3 (1 : 1000) at 4°C overnight. Membranes were washed and incubated in HRP-labeled secondary antibodies at 20–25°C. The blots were visualized using chemiluminescent detection. The relative intensities of each protein band were determined using GAPDH as an internal reference.

### 2.13. Statistics

Differences between groups were analyzed using one-way ANOVA, the Tukey-Kramer multiple comparison test, and the Duncan comparison test. All statistical analyses were performed using GraphPad Prism 7.00. Statistical differences were considered significant at *p* < 0.05.

## 3. Results

### 3.1. Curcumin Reverses the Phenotype of PCOS Model Rats

**Figure 1:**
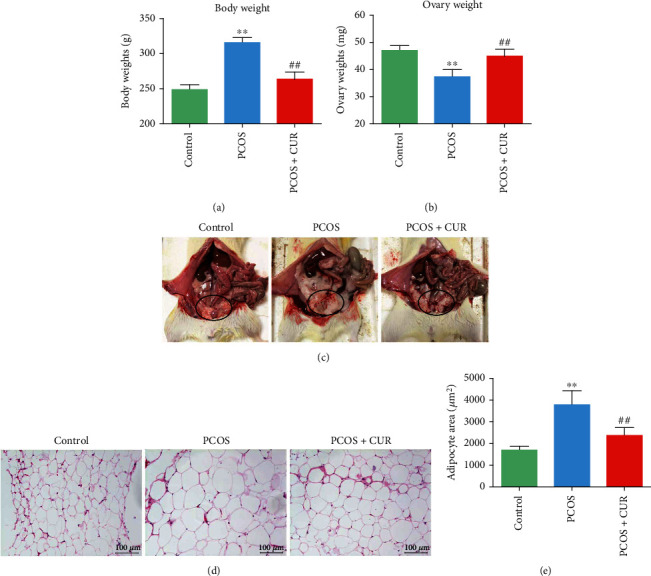
The curcumin effects on body weight, ovarian weight, and abdominal fat in DHEA-induced PCOS-like rats. PCOS-like rats were induced with DHEA-exposed, together with or without curcumin treatment. (a, b) Rat body weight and ovarian weight were measured in all experimental groups after 8 weeks of curcumin treatment. (c) A macroscopic view of the abdominal fat of each group of rats was shown. (d, e) Abdominal fat in all experimental group rats was assessed by H&E staining (20x) and the quantitative analysis. Three independent experiments were performed with similar results. Data are shown as the mean ± SEM. ^∗∗^*p* < 0.05 vs. control group; ^##^*p* < 0.05 vs. PCOS group.

Changes in the estrus cycle were monitored by vaginal exfoliated cell smears of rats (Supplemental Figure 1A). Analysis of the smears showed that the preestrus smears mainly contained small, round, and nucleated epithelial cells (squamous epithelial cells); the estrus period is dominated by irregular keratinized squamous epithelial cells and the metestrus period is dominated by white blood cells and keratinocytes, while the diestrus period is characterized by the appearance of white blood cells and round epithelial cells. The estrus cycle of all rats in the experimental group was continuously observed for 10 days. The results showed that the rats in the PCOS group lost their regular estrus cycle. After the PCOS rats received curcumin, the estrus cycle returned to normal (Figure 1(b)). In addition, DHEA-induction of vesicular follicle proliferation was substantially reduced after curcumin treatment, whereas the number of corpus lutea increased (Supplemental Figure 1C). ELISA was used to detect the levels of serum hormones T, LH, and FSH in all experimental groups. The results showed that serum T and LH levels were markedly elevated, while serum FSH levels were decreased in the PCOS group compared with the control group; this effect was reversed in the curcumin treatment group (Supplemental Figure 1D).

Obesity, particularly visceral adiposity, which is common in both obese and nonobese women with PCOS, amplifies and worsens all metabolic and reproductive outcomes in PCOS [28]. In our study, compared with those of the control group, the weight of rats of the PCOS group was significantly higher and the abdominal fat content was prominently increased, but the ovarian weight was reduced. After the 8 weeks of curcumin treatment for rats of the PCOS group, their weight was markedly decreased, abdominal fat content was reduced, and ovarian weight was restored (Figures 1(a)–1(c)). Moreover, H&E staining revealed that the rats with DHEA-induced PCOS had larger adipocyte areas than the control group rats; this effect was reversed by curcumin treatment (Figures 1(d) and 1(e)).

### 3.2. Curcumin Attenuates ER Stress in Primary Cultured GCs

**Figure 2:**
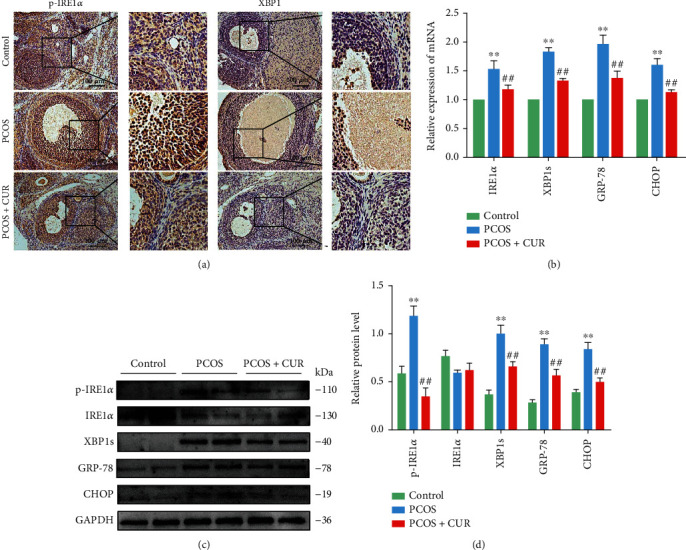
Curcumin relieves the ER stress strongly activated by DHEA in the ovarian tissue of PCOS-like rats. (a) The levels of p-IRE1*α* and XBP1 were measured with immunohistochemical staining. (b) The expression of IRE1*α*, XBP1, GRP-78, and CHOP in the ovaries was assessed by qRT-PCR assay. (c, d) The protein expression of p-IRE1*α*, IRE1*α*, XBP1, GRP-78, and CHOP in ovarian tissues was assessed with western blotting. Representative data are displayed, and the relative protein intensity of p-IRE1*α*, IRE1*α*, XBP1, GRP-78, and CHOP was normalized to GAPDH. Three independent experiments were performed with similar results. Data are shown as the mean ± SEM. ^∗∗^*p* < 0.05 vs. control group; ^##^*p* < 0.05 vs. PCOS group.

**Figure 3:**
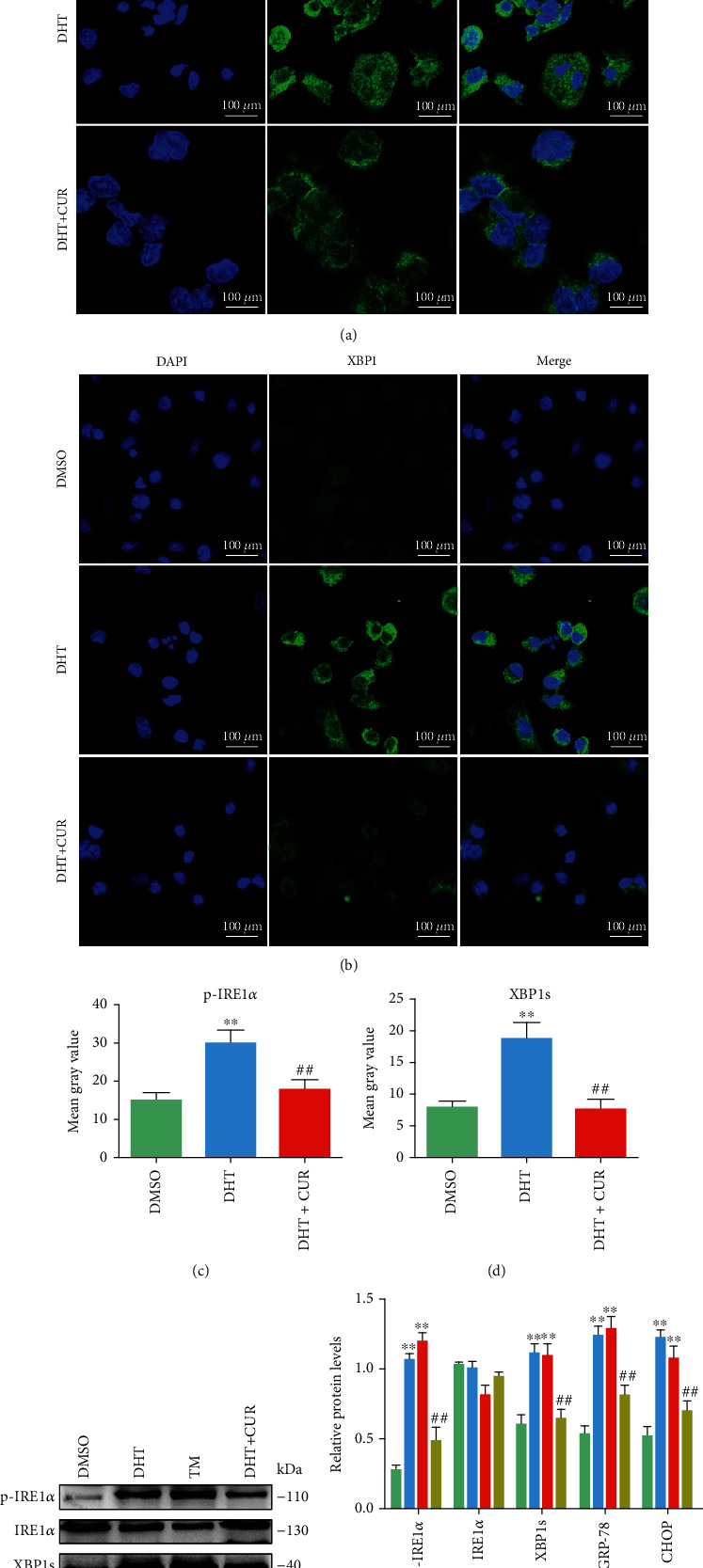
Curcumin inhibits the ER stress and the IRE1*α*-XBP1 pathway activated by DHT in the ovarian GCs. Curcumin-treated GCs were induced by DHT/TM. (a–d) Round coverslips of ovarian GCs were immunostained for revealing p-IRE1*α* and XBP1 expression levels (green) as indicated. The slides were counterstained with DAPI (blue), and the images were captured using an inverted fluorescence microscope. Three independent experiments were performed with similar results. Data are shown as the mean ± SEM. ^∗∗^*p* < 0.05 and ^∗∗^*p* < 0.05 vs. DMSO group; ^##^*p* < 0.05 vs. DHT group.

Research has shown that ER stress and UPR activation are increased in GCs of PCOS patients [16]. To confirm that the ER stress-related IRE1*α*-XBP1 pathway was activated in the ovary of PCOS model rats, immunofluorescence was used to analyze the distribution and intensity of p-IRE1*α* and XBP1. Results showed that these markers were more strongly expressed in the ovaries of PCOS model rats than in those of the control group. Furthermore, western blot analysis and qRT-PCR showed that the levels of p-IRE1*α*, XBP1, GRP78, and CHOP were higher in the ovaries of PCOS model rats than in those of control rats. As expected, the ovarian tissue in PCOS+CUR group rats showed notably reduced levels of p-IRE1*α*, XBP1, GRP78, and CHOP expression (Figures 2(a)–2(d)). Tunicamycin (TM) is commonly used to create ER stress models [29], and we used TM to induce ER stress in rat GCs to be used as a positive control. To verify these results, we measured the expression of ER stress-related IRE1*α*-XBP1 pathway proteins using immunofluorescence and western blot analysis in vitro. Higher p-IRE1*α*, XBP1, GRP78, and CHOP levels were observed in the DHT group than in the control group, and the DHT-induced elevations in p-IRE1*α*, XBP1, GRP78, and CHOP protein levels in GCs were attenuated by curcumin (Figures 3(a)–3(f)).

### 3.3. Curcumin Reverses DHEA-Induced ER Stress by Activating the PI3K/AKT Pathway

**Figure 4:**
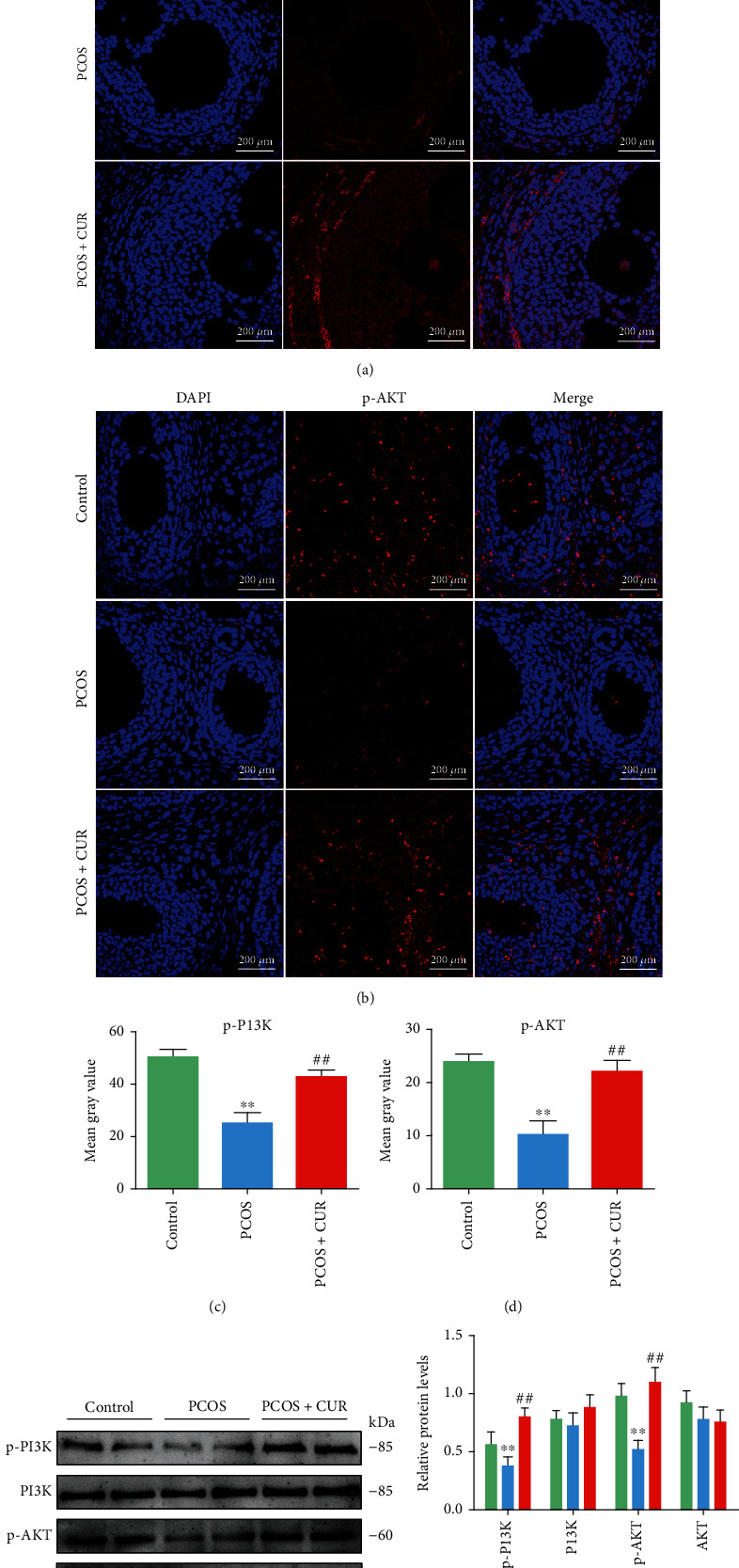
Curcumin activates the PI3K/AKT pathway inhibited by DHEA in ovary tissues of PCOS-like rats. (a–d) Sections of ovarian tissues were immunostained for revealing p-PI3K/p-AKT (red) as indicated. The slides were counterstained with DAPI (blue), and the images were captured using an inverted fluorescence microscope. (e, f) The protein expression of p-PI3K, PI3K, AKT, and p-AKT in ovarian tissues was assessed with western blotting. Representative data are displayed, and the relative protein intensity of p-PI3K, PI3K, AKT, and p-AKT was normalized to GAPDH. Three independent experiments were performed with similar results. Data are shown as the mean ± SEM. ^∗∗^*p* < 0.05 vs. control group; ^##^*p* < 0.05 vs. PCOS group.

IR is prevalent in women with PCOS [14], and the PI3K/AKT signaling pathway is closely linked to the occurrence and development of IR [30]. Therefore, to explore the mechanisms by which curcumin alleviates ER stress in ovarian tissues, the expressions of proteins involved in the PI3K/AKT signaling were determined by immunofluorescence, western blotting, and immunohistochemistry. In the PCOS group, the expression levels of p-PI3K and p-AKT were significantly lower than those in the control group, while the protein levels of p-PI3K and p-AKT in the PCOS+CUR group were significantly higher than those in the PCOS group (Figures 4(a)–4(f)) (Supplemental Figure 2A).

In addition, the effects of curcumin treatment on primary cultured GCs were determined in vitro. GC cell viability was significantly improved by treatment with 20 *μ*M curcumin (Supplemental Figure 3A). Furthermore, the primary GCs were treated with DHT, H_2_O_2_, and 20 *μ*M curcumin, and the increase in ROS induced by DHT was consistent with that observed in the positive control upon H_2_O_2_ treatment. Curcumin treatment significantly reduced the level of ROS in GCs (Supplemental Figures 3B and 3C).

**Figure 5:**
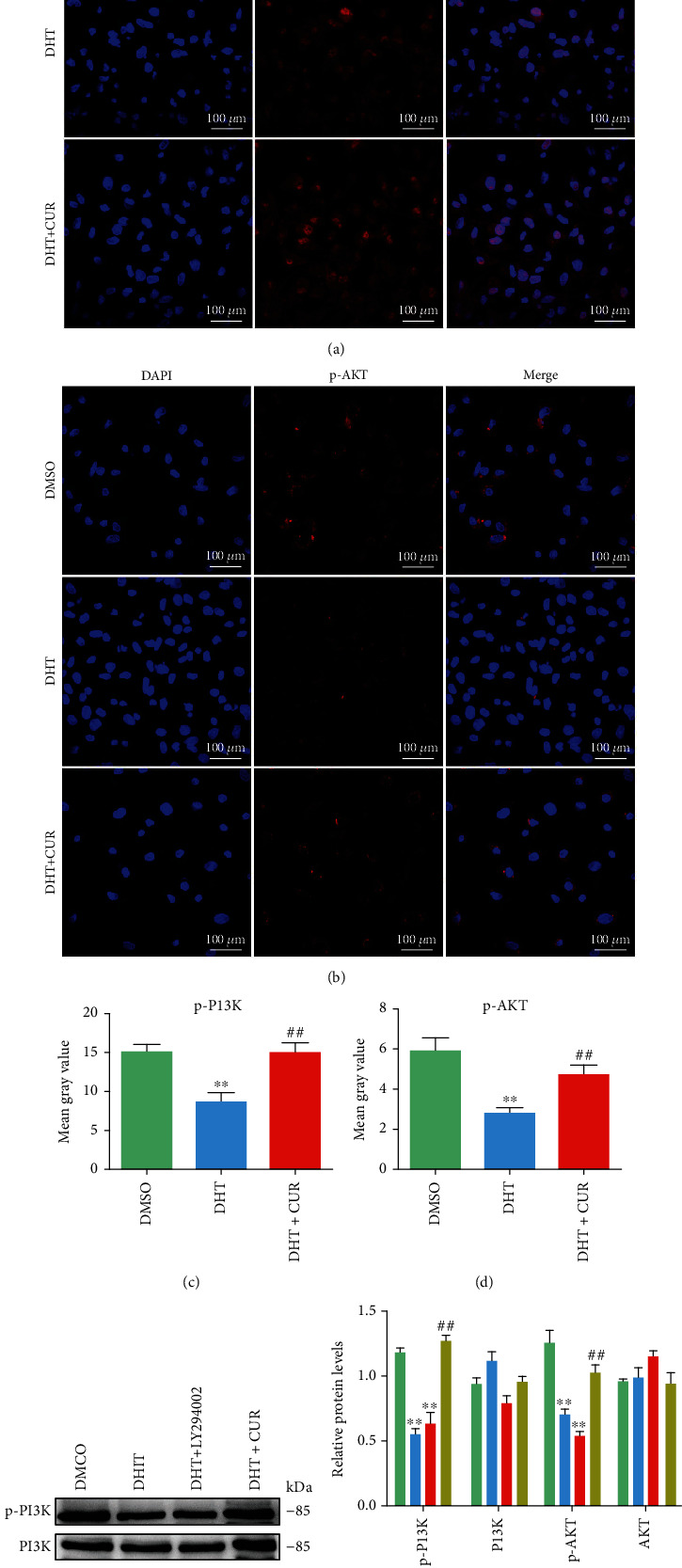
Curcumin activates the PI3K/AKT pathway inhibited by DHT in ovary GCs. Curcumin/LY294002-treated GCs were induced by DHT. (a–d) Round coverslips of ovarian GCs were immunostained for revealing p-PI3K and p-AKT expression levels (red) as indicated. The slides were counterstained with DAPI (blue), and the images were captured using an inverted fluorescence microscope. (e, f) The protein expression of p-PI3K, PI3K, AKT, and p-AKT in ovarian GCs was assessed with western blotting. Representative data are displayed, and the relative protein intensity of p-PI3K, PI3K, AKT, and p-AKT was normalized to GAPDH. Three independent experiments were performed with similar results. Data are shown as the mean ± SEM. ^∗∗^*p* < 0.05 vs. DMSO group; ^##^*p* < 0.05 vs. DHT group.

**Figure 6:**
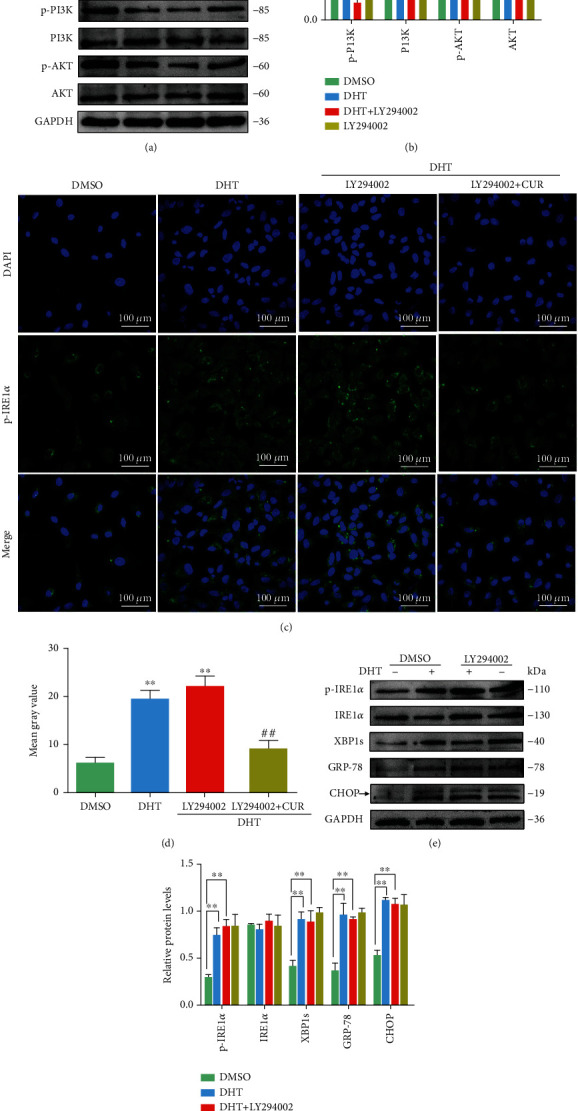
Ameliorative effects of curcumin on DHT-induced ER stress in ovarian GCs mediated via increasing of PI3K/AKT phosphorylation. (a, b) Western blot results showing the changes in the indicated proteins in GCs treated with DHT after incubation with LY294002 (a PI3K inhibitor). (c, d) The p-IRE1*α* protein level in GCs treated with DHT, LY294002, and curcumin. (e, f) Changes in ER stress-related protein levels in GCs treated with DHT and LY294002 as detected by western blot. Three independent experiments were performed with similar results. Data are shown as the mean ± SEM. ^∗∗^*p* < 0.05 and ^∗∗∗∗^*p* < 0.01. ^∗∗^*p* < 0.05 vs. DMSO group; ^##^*p* < 0.05 vs. DHT/DHT+LY294002 group.

LY294002, an inhibitor of PI3K, was used to confirm that PI3K plays a key role in DHT-induced ER stress activation. Based on immunofluorescence and western blotting assays, the expression of p-PI3K and p-AKT in GCs was inhibited by DHT induction, whereas their expression was significantly increased after curcumin treatment; this result is consistent with those obtained from the in vivo studies (Figures 5(a)–5(f)). The levels of p-PI3K and p-AKT in DHT-treated GCs showed no difference after LY294002 treatment (Figures 6(a) and 6(b)). GCs were treated with DHT for 24 h, followed by LY294002 (10 *μ*M, HY-10108, Shanghai, China) for 24 h, and then curcumin for further 24 h. The expression level of the ER stress protein p-IRE1*α* was detected by immunofluorescence staining. As expected, LY294002 aggravated the level of p-IRE1*α* in DHT-treated GCs, which was reversed by curcumin treatment (Figures 6(c) and 6(d)). Furthermore, treatment of GCs with LY294002 also aggravated the DHT-induced increase in the ER stress-related proteins p-IRE1*α*, XBP1, GRP78, and CHOP (Figures 6(e) and 6(f)).

**Figure 7:**
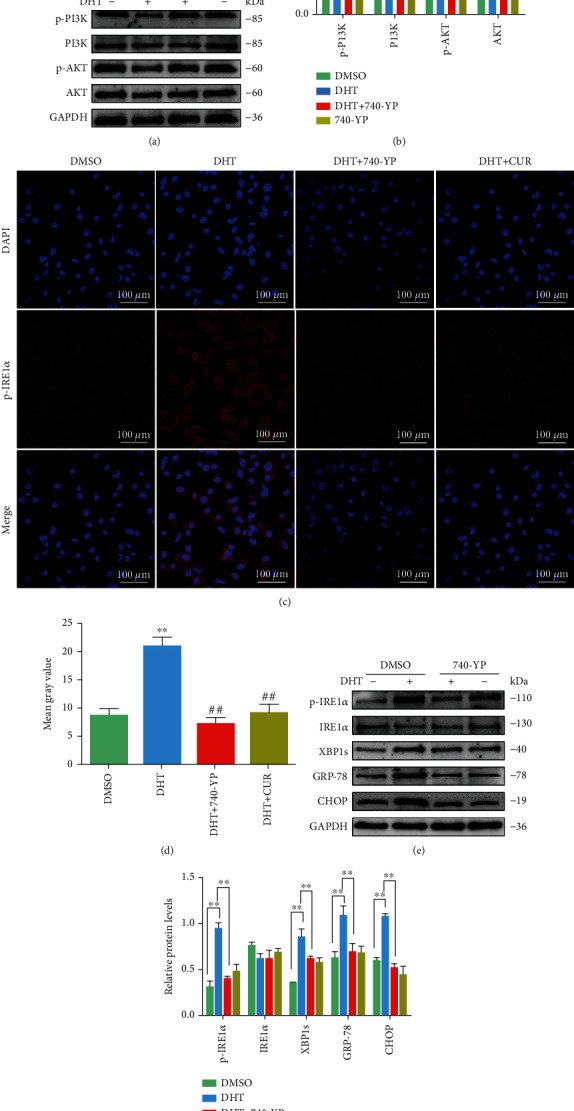
Effects of curcumin on DHT-induced ER stress in ovarian GCs mediated via increasing of PI3K/AKT phosphorylation. (a, b) Western blot results showing the changes of the p-PI3K, PI3K, p-AKT, and AKT levels in GCs treated with DHT and 740-YP (a PI3K activator). (c, d) The p-IRE1*α* expression level in GCs treated with DHT, 740-YP, and curcumin by immunofluorescence staining (60x). (e, f) Changes in ER stress-related protein p-IRE1*α*, IRE1*α*, XBP1, GRP-78, and CHOP levels in GCs treated with DHT and 740-YP as detected by western blot. Three independent experiments were performed with similar results. Data are shown as the mean ± SEM. ^∗∗^*p* < 0.05 and ^∗∗^*p* < 0.05 vs. DMSO group; ^##^*p* < 0.05 vs. DHT group.

740 Y-P is an effective PI3K activator that strongly modulates the PI3K/AKT signal transduction pathway [31]. GCs were incubated with 740-YP (20 *μ*M, HY-P0175, Shanghai, China) for 24 h after DHT induction. 740-YP activated the PI3K/AKT signaling pathway, which was inhibited by DHT in GCs (Figures 7(a) and 7(b)). In addition, DHT-induced activation of p-IRE1*α* levels in GCs was reduced by 740-YP treatment. This is consistent with the observation that curcumin treatment inhibited the high expression of DHT-induced p-IRE1*α* protein in GCs (Figures 7(c) and 7(d)). DHT-induced elevations in p-IRE1*α*, XBP1, GRP78, and CHOP protein levels in GCs were also decreased upon 740-YP treatment (Figures 7(e) and 7(f)).

### 3.4. Curcumin Protects against the Apoptosis of GCs, Possibly by Inhibiting IRE1*α*-XBP1 Levels and Activating the PI3K/AKT Pathway

To further clarify the protective mechanism of curcumin on GC apoptosis in hyperandrogen-induced PCOS model rats, we measured ovarian tissue and GC apoptosis using several methods. AKT can affect the activity of p53 by phosphorylation of the p53-binding protein MDM2. Phosphorylated MDM2 translocates to the nucleus and binds to p53, triggering its degradation and thus affecting cell survival. In addition, AKT can activate IKK and cross-talk with the NF-*κ*B pathway. Therefore, the effects of curcumin treatment on the p53 and NF-*κ*B signaling pathways in the ovaries of PCOS-like rats were analyzed by immunohistochemistry and western blotting. The results showed that curcumin significantly reduced the high expression levels of p-p53, p-NF-*κ*B, and p-JNK in the ovaries of DHEA-induced PCOS model rats (Supplemental Figures 2B–2D). TUNEL analysis was performed on ovarian sections. Curcumin treatment significantly reduced the number of TUNEL-positive cells in the ovarian tissues of PCOS model rats (Figures 4(a) and 4(b)). The expression levels of apoptosis-related proteins (caspase-12, caspase-9, cleaved-caspase-3, Bad, Bax, and Bcl-2) were significantly increased in the PCOS group, whereas the expression of these proteins was markedly decreased in ovarian tissue after curcumin treatment (Supplemental Figures 4C–4F).

**Figure 8:**
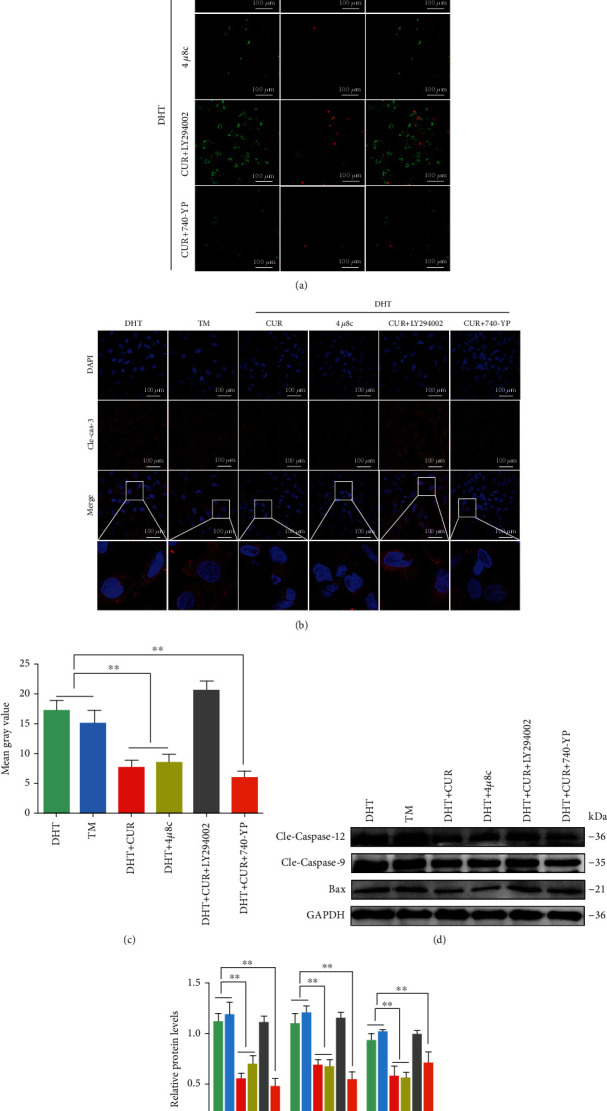
Curcumin protects hyperandrogen-induced apoptosis in ovarian GCs in PCOS rats by inhibiting the IRE1*α*-XBP1 pathway and activating the PI3K/AKT signal. DHT/TM-induced GCs were treated with curcumin, 4u8c, LY294002, and 740-YP with or without DHT. (a) GCs were incubated with Annexin V-FITC and PI. The cells were imaged for apoptosis detection using a FV3000 Olympus microscope. (b, c) Apoptosis protein expression of cleaved-caspase-3 was analyzed by immunofluorescence staining (60x) and quantified using ImageJ software. (d, e) The expression of casapase-12, caspase-9, and Bax was assessed by the western blot assay. Three independent experiments were performed with similar results. Data are shown as the mean ± SEM. ^∗∗^*p* < 0.05.

Next, primary cultured GCs were treated with DHT, TM, curcumin, 4*μ*8c, LY294002, and 740-YP for 48 h and subsequently analyzed by PI and FITC-Annexin V staining followed by flow cytometry and microscopic examination. The apoptosis rate was higher in DHT/TM/LY294002-treated GCs. Curcumin, 4u8c, and 740-YP slightly attenuated this increase (Supplemental Figure 3D) (Figure 8(a)). The results of immunofluorescence and western blotting assays revealed the same effect; the high expression levels of cleaved-caspase-3, caspase-12, caspase-9, and Bax in GCs treated with DHT/TM/LY294002 were reversed after treatment with curcumin, 4u8c, and 740-YP. These results suggest that curcumin alleviates GC apoptosis by inhibiting the ER stress-related IRE1*α*-XBP1 pathway and upregulating the PI3K/AKT signaling pathway (Figures 8(b)–8(e)).

## 4. Discussion

Excess androgens and ovarian dysfunction are the main symptoms of PCOS. The long-term effects of a continuous, hyperandrogenic environment include oxidative stress, low-grade inflammation, mitochondrial dysfunction, ovarian interstitial cell fibrosis, and ER stress. In our study, DHEA was continuously injected subcutaneously into adolescent rats for 35 days. The rats lost their regular estrus cycle, showing disordered serum hormone levels and pathological changes in the ovaries. Clinical PCOS patients have high serum T levels, and the ovaries present a high androgenic follicular environment. Therefore, it is necessary to study the effects of hyperandrogenism in the follicles. Ovarian GCs are critical in reproduction. Their proliferation and secretion functions are closely related to the growth and maturation of oocytes. Research has shown that androgen receptor (AR) mRNA expression was significantly higher in the GCs of patients with higher androgen levels in the follicular fluid than in those of control patients [32]. This suggests that the increase in AR expression did not enhance the function of transforming androgens. Thus, the mechanism underlying the influence of the hyperandrogen environment on GCs remains unclear. Several studies have shown that T-induced ER stress leads to apoptosis in human and mouse cumulus cells [33]. Another study reported that the apoptosis rates and signals of ovarian GCs induced by T increased slightly, but not significantly [16]. However, in our study, DHT-induced primary GCs showed obvious apoptosis (Figures 4 and 8). Because DHT is a steroid hormone, T is produced under the action of 5-*α* reductase (5*α*-R); therefore, we speculate that the insignificant apoptosis of GCs induced by T may be related to abnormal levels of intracellular 5*α*-R in ovarian GCs. However, whether this is related to the long-term hyperandrogenic environment of follicles requires further study.

ER stress-mediated apoptosis in GCs may be related to the pathogenesis of PCOS. Therefore, understanding the role of ER stress in the pathogenesis of PCOS has important clinical significance in the prevention and treatment of PCOS. Studies have shown that long-term excessive ER stress in GCs impairs protein secretion, mitochondrial activity, and the formation of oocyte developmental competence, leading to GC dysfunction that indirectly impairs the ability of oocytes [34]. The IRE1*α*-XBP1 signaling pathway is an evolutionarily conserved branch of the UPR, which controls various immune metabolic processes while maintaining homeostasis of the endoplasmic reticulum. In addition, the IRE1*α*-XBP1 pathway is involved in insulin and glucagon secretion by regulating pancreatic *α/β*-cell functions, thus controlling glucose homeostasis [35]. IR and hyperinsulinemia are the basic features of abnormal glucose metabolism in patients with PCOS. Studies have also reported that the activity of the insulin receptor, insulin receptor substrate 1 (IRS-1), PI3K, and glucose transporter 4 was reduced in PCOS patients [36]. In our study, hyperandrogenism induced ER stress and IRE1*α*-XBP1 pathway activation in ovarian GCs of PCOS model rats. Furthermore, the expression levels of p-PI3K and p-AKT in the PCOS group were significantly lower than those in the control group. We speculate that hyperandrogenism leads to GC dysfunction, which may be related to ER stress and PI3K/AKT pathway inhibition caused by the ovarian hyperandrogen environment.

With reported antioxidant, anti-inflammatory, antimicrobial, antidiabetic, and other physiological activities in different disease models, curcumin has great potential for health applications. In a randomized double-blind placebo-controlled trial, individuals with PCOS were treated with curcumin (500 mg, three times daily) for 12 weeks. The results showed that curcumin significantly reduced the amount of DHEA and might even increase estradiol levels in women with PCOS [37]. Another report showed that treatment with curcumin-encapsulated arginine and N-acetyl histidine-modified chitosan (Arg-CS-NAcHis/Cur) nanoparticles reversed many symptoms of PCOS [37]. Our study showed that curcumin protected ovarian GCs from hyperandrogen-induced apoptosis in PCOS model rats probably by inhibiting the IRE1*α*-XBP1 levels and activating the PI3K/AKT signaling pathway. These observations indicate that curcumin might be a safe and beneficial supplement for patients with PCOS. However, further in-depth trials investigating different dosages over longer durations are needed to confirm these findings.

## Figures and Tables

**Table 1:** Sequences of primers designed for RT-qPCR.

Genes	Forward	Reverse	Length
*IRE-1α*	5′-AACACACCGACCACCGTATC-3′	5′-AGGGTACTGGGTAAGGTCTC-3′	282
*XBP1s*	5′-TAGAAAGAAAGCCCGGATGA-3′	5′-TCTCAATCACAAGCCCATGA-3′	122
*GRP-78*	5′-GCCAACTGTAACAATCAA-3′	5′-GCTGTCACTCGGAGAATA-3′	165
*CHOP*	5′-GCTGGAAGCCTGGTATG-3′	5′-CTTTGGGATGTGCGTGT-3′	183
*GAPDH*	5′-ACTCACTCTTCTACCTTTGATGCT-3′	5′-TGTTGCTGTAGCCAAATTCA-3′	100

**Table 2:** Reaction conditions of RT-qPCR.

Stage 1	Predegeneration	Rep: 1	95°C	30 sec
Stage 2	Circular reaction	Reps: 40	95°C	10 sec
			60°C	30 sec
Stage 3	Melting curve	Rep: 1	95°C	15 sec
			60°C	60 sec
			95°C	15 sec

## Data Availability

The datasets generated and/or analyzed during the current study are available from the corresponding authors on reasonable request. In addition, all data generated or analyzed during this study are included in this published article.

## Supplementary Materials

Supplementary Materials Supplementary data: Supplementary data are available at *Oxidative Medicine and Cellular Longevity* online. Supplemental Figure 1: curcumin treatment reverses the phenotype of PCOS-like rats. (A) The characteristics of the smears at different stages of estrus. (B) The estrus cycle of all rats in the experimental group was showed. (C) Ovarian and follicular morphology was assessed by H&E staining. (D) The hormones T, LH, and FSH levels were analyzed using enzyme-linked immunosorbent assay kits. Three independent experiments were performed and yielded similar results. Data are shown as the mean ± SEM. ^∗∗^*p* < 0.05 vs. control group; ^##^*p* < 0.05 vs. PCOS group. Supplemental Figure 2: curcumin activates the PI3K/AKT pathway in the ovarian tissue of PCOS model rats. (A) The expression levels of p-PI3K and p-AKT in ovarian tissues of PCOS-like rats were detected using immunofluorescence. (B) Immunofluorescence staining of p-p53 and p-NF-*κ*B in ovarian sections from the mice in three different groups. (C, D) The expressions of p-p53, p-NF-*κ*B, NF-*κ*B, p-JNK, and JNK were assessed using western blotting. Three independent experiments were performed and yielded similar results. Data are shown as the mean ± SEM. ^∗∗^*p* < 0.05 vs. control group; ^##^*p* < 0.05 vs. PCOS group. Supplemental Figure 3: the protective effects of curcumin on cell viability and ROS in DHT-induced ovarian GCs. (A) Cell viability of GCs after DHT and curcumin treatment was analyzed using CCK-8 kits. (B, C) ROS generation in GCs following DHT/H_2_O_2_ and different concentrations of curcumin treatment was measured using the DCF-DA probe. DCF-DA fluorescence (green fluorescence) was measured using a confocal microscopy (20x). (D) FITC-Annexin V and propidium iodide (PI) staining of GCs and flow cytometry analysis of GCs to determine the apoptotic rates after treatment with curcumin, 4*μ*8c, LY294002, and 740-YP with or without DHT. Three independent experiments were performed and yielded similar results. Data are shown as the mean ± SEM. ^∗∗^*p* < 0.05. Supplemental Figure 4: analysis of apoptosis in ovarian tissues of all experimental groups. (A, B) TUNEL analysis and measurement of the ovarian sections of the control, PCOS-like, and the PCOS+CUR groups. (C–F) The apoptotic protein expressions of Bad, Bax, Bcl-2, caspase-12, caspase-9, and cleaved-caspase-3 in ovarian tissues were assessed using western blotting. Representative data are displayed, and the relative protein intensities of Bad, Bax, Bcl-2, caspase-12, caspase-9, and cleaved-caspase-3 were normalized to GAPDH. Three independent experiments were performed and yielded similar results. Data are shown as the mean ± SEM. ^∗∗^*p* < 0.05 vs. control group; ^##^*p* < 0.05, vs. PCOS group.
