# Supplementary material for: Curcumin Inhibits Hyperandrogen-Induced IRE1α-XBP1 Pathway Activation by Activating the PI3K/AKT Signaling in Ovarian Granulosa Cells of PCOS Model Rats
Source: Oxid Med Cell Longev. 2022 Aug 24;2022:2113293. doi: 10.1155/2022/2113293 (PMC9433213; doi:10.1155/2022/2113293)
Supplement: Supplementary Materials — Supplementary data: Supplementary data are available at Oxidative Medicine and Cellular Longevity online. Supplemental Figure 1: curcumin treatment reverses the phenotype of PCOS-like rats. (A) The characteristics of the smears at different stages of estrus. (B) The estrus cycle of all rats in the experimental group was showed. (C) Ovarian and follicular morphology was assessed by H&E staining. (D) The hormones T, LH, and FSH levels were analyzed using enzyme-linked immunosorbent assay kits. Three independent experiments were performed and yielded similar results. Data are shown as the mean ± SEM. ∗∗p < 0.05 vs. control group; ##p < 0.05 vs. PCOS group. Supplemental Figure 2: curcumin activates the PI3K/AKT pathway in the ovarian tissue of PCOS model rats. (A) The expression levels of p-PI3K and p-AKT in ovarian tissues of PCOS-like rats were detected using immunofluorescence. (B) Immunofluorescence staining of p-p53 and p-NF-κB in ovarian sections from the mice in three different groups. (C, D) The expressions of p-p53, p-NF-κB, NF-κB, p-JNK, and JNK were assessed using western blotting. Three independent experiments were performed and yielded similar results. Data are shown as the mean ± SEM. ∗∗p < 0.05 vs. control group; ##p < 0.05 vs. PCOS group. Supplemental Figure 3: the protective effects of curcumin on cell viability and ROS in DHT-induced ovarian GCs. (A) Cell viability of GCs after DHT and curcumin treatment was analyzed using CCK-8 kits. (B, C) ROS generation in GCs following DHT/H2O2 and different concentrations of curcumin treatment was measured using the DCF-DA probe. DCF-DA fluorescence (green fluorescence) was measured using a confocal microscopy (20x). (D) FITC-Annexin V and propidium iodide (PI) staining of GCs and flow cytometry analysis of GCs to determine the apoptotic rates after treatment with curcumin, 4μ8c, LY294002, and 740-YP with or without DHT. Three independent experiments were performed and yielded similar results. Data are shown as th [file 2113293.f1.docx]

**Supplementary material**

Sup-Figure 1

**
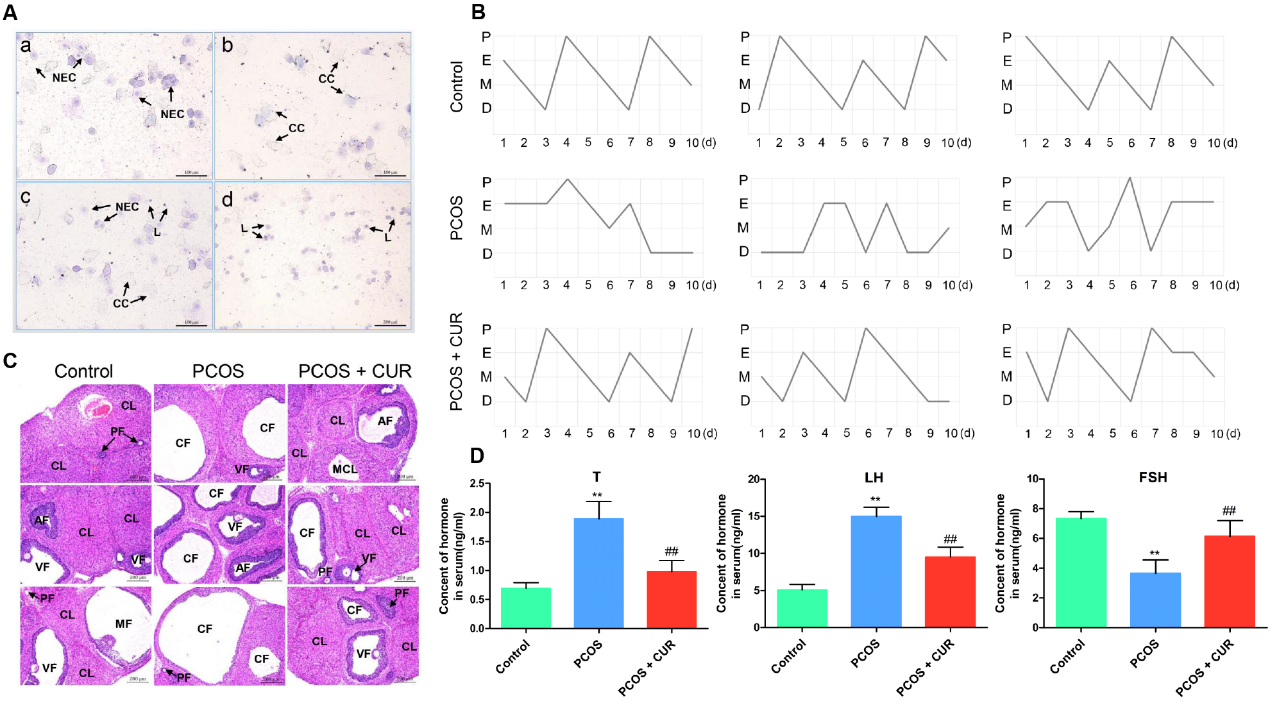
**

**Sup-Figure 1: Curcumin treatment reverses the phenotype of PCOS-like rats**

(A) The characteristics of the smears at different stages of estrus. (B) The estrus cycle of all rats in the experimental group was continuously showed. (C) Ovarian and follicular morphology was assessed by H&E staining. (D) The hormone T, LH and FSH levels were analyzed using enzyme-linked immunosorbent assay kits. Three independent experiments were performed with similar results. Data are shown as mean ± SEM. ** *P* < 0.05, vs. control group; ## *P* < 0.05, vs. PCOS group.

Sup-Figure 2


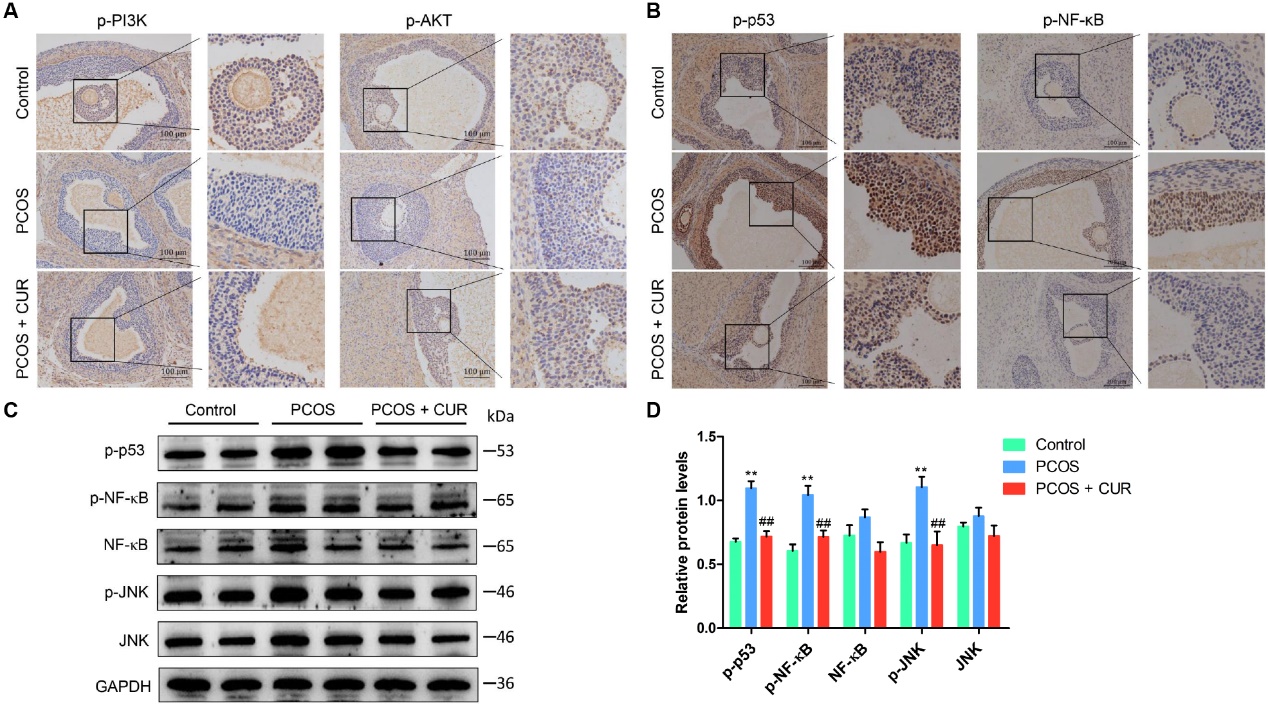


**Sup-Figure 2: Curcumin activates the PI3K/AKT pathway in the ovarian tissue of PCOS model rats**

(A) The expression level of p-PI3K, p-AKT in ovarian tissues of PCOS-like rats was detected by immunofluorescence. (B) Immunofluorescence staining of p-p53, and p-NF-κB in ovarian sections from the mice in three different groups. (C, D) The expression of p-p53, p-NF-κB, NF-κB, p-JNK and JNK were assessed by western blot assay. Three independent experiments were performed with similar results. Data are shown as mean ± SEM. ** *P* < 0.05, vs. control group; ## *P* < 0.05, vs. PCOS group.

Sup-Figure 3


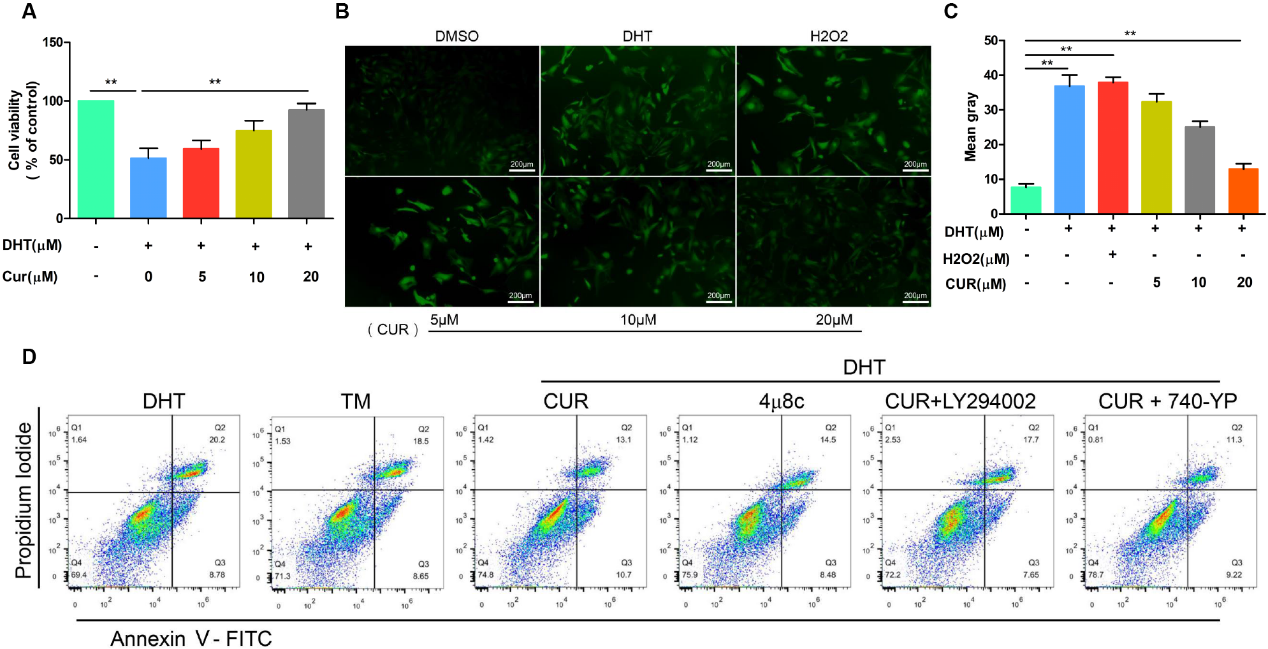


**Sup-Figure 3: The protective effects of curcumin on cell** [**viability**](javascript:;)**, and ROS in DHT-induced ovarian GCs.**

(A) Cell viability of GCs after DHT and curcumin treated was analyzed by CCK-8 kits. (B, C) ROS generation in GCs following [DHT/H_2_O_2_](javascript:;) and different concentrations of curcumin treatment was measured using the DCF-DA probe. DCF-DA fluorescence (green fluorescence) was measured by confocal microscopy (20×). (D) FITC-Annexin V and propidium iodide (PI) staining of GCs and flow cytometry analysis of GCs apoptotic rates after treatment with curcumin, 4u8c, LY294002, and 740-YP with or without DHT. Three independent experiments were performed with similar results. Data are shown as mean ± SEM. ** *P* < 0.05.

Sup-Figure 4


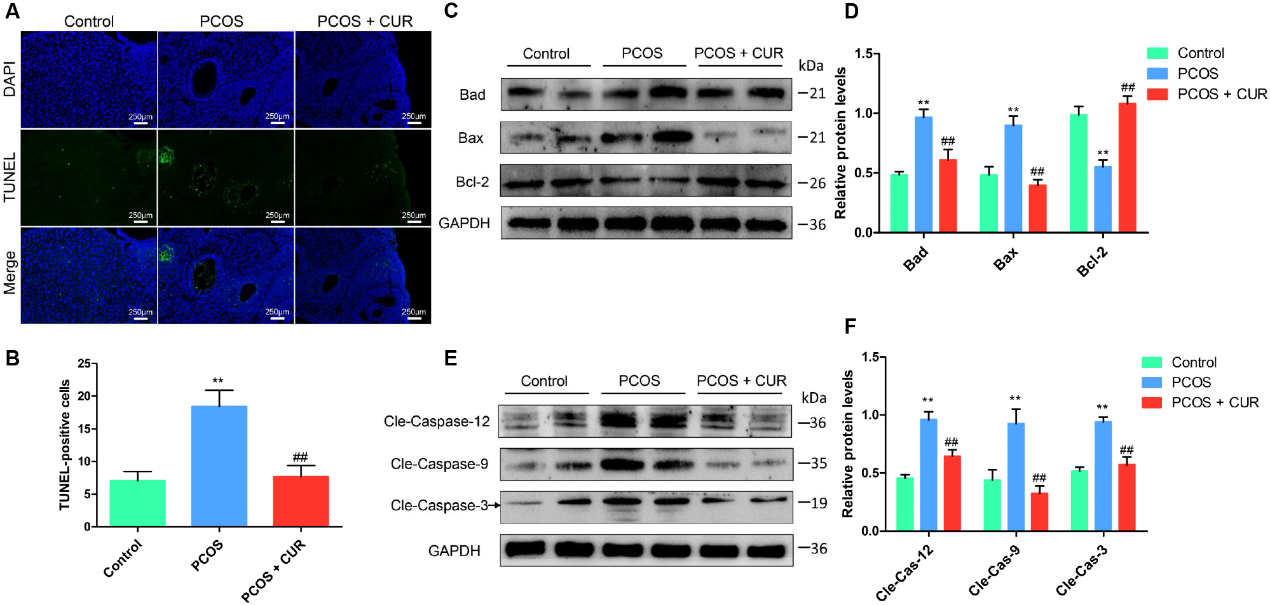


**Sup-Figure 4: Analysis of apoptosis in ovarian tissues of all experimental groups.**

(A, B) TUNEL analysis and measured on ovarian sections of control, PCOS-like and PCOS + CUR groups. (C-F) The apoptosis protein expression of Bad, Bax, Bcl-2, Caspase-12, Caspase-9, and Cleaved-caspase-3 in ovarian tissues was assessed with western blotting. Representative data are displayed and the relative protein intensity of Bad, Bax, Bcl-2, Caspase-12, Caspase-9, and Cleaved-caspase-3 was normalized to GAPDH. Three independent experiments were performed with similar results. Data are shown as mean ± SEM. ** *P* < 0.05, vs. control group; ## *P* < 0.05, vs. PCOS group.
